# Association of T3/T4 ratio with inflammatory indicators and all-cause mortality in stroke survivors

**DOI:** 10.3389/fendo.2024.1509501

**Published:** 2025-01-08

**Authors:** Sheng Zhang, Zhongzhou Su, Xianqiang Wen

**Affiliations:** Department of Neurosurgery, Huzhou Central Hospital, Huzhou, China

**Keywords:** NHANES, stroke, T3/T4 ratio, NPAR, mediation effect, forest plot

## Abstract

**Background:**

Abnormal thyroid hormone levels may occur in critical illness, which may have an interactive relationship with inflammatory reaction. At present, the relationship between triiodothyronine (T3)/thyroxine (T4) ratio and inflammatory indicators and all-cause mortality of stroke survivors is still unclear.

**Methods:**

We obtained the relevant data of the respondents from 2007 to 2012 through the National Health and Nutrition Examination Survey (NHANES) database for statistical analysis. The ratio of T3/T4, a continuous variable, is transformed into three groups of classified variables, namely Q1, Q2 and Q3. The relationship between T3/T4 ratio and mortality was analyzed by Log-Rank test and K-M survival curve. Pearson correlation analysis was used to analyze the correlation between T3/T4 ratio and white blood cell (WBC), Neutrophil-to-Lymphocyte Ratio (NLR), systemic immune-inflammation index (SII) and neutrophil percentage-to-albumin ratio (NPAR). Cox univariate and multivariate analysis was used to identify independent risk factors for all-cause mortality in stroke survivors and a nomogram was drawn. Restricted cubic spline (RCS) curve was drawn to determine whether there was a linear relationship between T3/T4 ratio and mortality and the best cut-off value. Subgroup analysis showed the difference between the T3/T4 ratio and all-cause mortality among subgroups and a forest plot was drawn. The mediation effect analysis was used to analyze whether the ratio of T3/T4 could mediate the survival time through inflammatory indicators.

**Results:**

According to the inclusion and exclusion criteria, a total of 267 people were included in the study, with a mortality rate of 49.06% (131/267), an average survival time of 111.22 ± 3.19 months, and a median survival time of 130 ± 11.27 months. The Log-Rank test and K-M survival curve showed that there were statistical differences among the Q1, Q2, and Q3 groups of the T3/T4 ratio (*x* ^2^ = 16.32, *p*<0.001), and the lower the T3/T4 level, the shorter the survival time. Pearson correlation analysis showed that the T3/T4 ratio had a linear relationship with NLR, SII, and NPAR, and only had a weak correlation with NPAR (*r* = -0.31, *p*<0.001). Cox univariate analysis showed that age, marital status, race, cancer, T3/T4 ratio, NPAR and all-cause mortality were related. Multivariate regression analysis showed that age ≥ 60 years, race of non-Hispanic black, low T3/T4 ratio (*p* = 0.014, *HR* = 0.92, 95% *CI* = 0.87~0.98) and high NPAR (*p* = 0.009, *HR* = 2.50, 95% *CI* = 1.26~4.99) were independent risk factors for all-cause mortality. The RCS curve shows that the ratio of T3/T4 is linearly correlated with mortality, and the optimal cutoff value of T3/T4 is 12.97. Subgroup analysis showed that T3/T4 ratio is more likely to affect the survival of stroke survivors with BMI 18.5~28. Mediation effect analysis showed that there was a mediation effect between T3/T4 ratio, NPAR and survival time. The effect size of T3/T4 directly affecting survival time is 78.45%, and the effect size of T3/T4 indirectly affecting survival time through NPAR is 21.55%.

**Conclusions:**

T3/T4 ratio is an independent risk factor for all-cause mortality in stroke survivors, especially in the people with BMI 18.5~28. T3/T4 ratio may mediate the survival time through NPAR level. Therefore, monitoring thyroid function is beneficial to the management of stroke survivors.

## Introduction

Euthyroid sick syndrome (ESS) is a well-known disease, which usually affects patients with many acute and chronic diseases. It is usually manifested by the decrease of serum T3, which is obviously related to morbidity and mortality (1, 2). Stroke is considered to be the main cause of adult disability and one of the main causes of death all over the world (3). The thyroid hormone in patients with acute stroke will also change abnormally, but the research results of the relationship between thyroid hormone level and clinical prognosis of patients with acute stroke are contradictory, and there is no consensus on the optimal target level in the course of disease (4).

T4 is the main product secreted by thyroid gland, and most of the biological activity T3 comes from the peripheral transformation from T4 to T3 (5). T3/T4 can reflect the conversion rate of thyroid hormone, which shows a predictive role in some diseases. Among the elderly patients with enterocolitis who received biological agents, people with high T3/T4 ratio had a higher cure rate (6). In subjects with normal thyroid function in pre-diabetes, low T3/T4 ratio is associated with hyperinsulinemia and insulin resistance (7). In animal model, the increase of T3/T4 ratio reduces the growth of prostate tumor (8). Generally speaking, in some diseases, low T3/T4 ratio is a bad signal, and its level may be related to inflammatory reaction. Therefore, this study collected the data of respondents in NHANES database from 2007 to 2012, and analyzed the relationship between T3/T4 ratio and inflammatory indicators and all-cause mortality in stroke survivors, so as to provide some insights for the management of stroke survivors.

## Methods

### Study population and ethics

**Figure 1:**
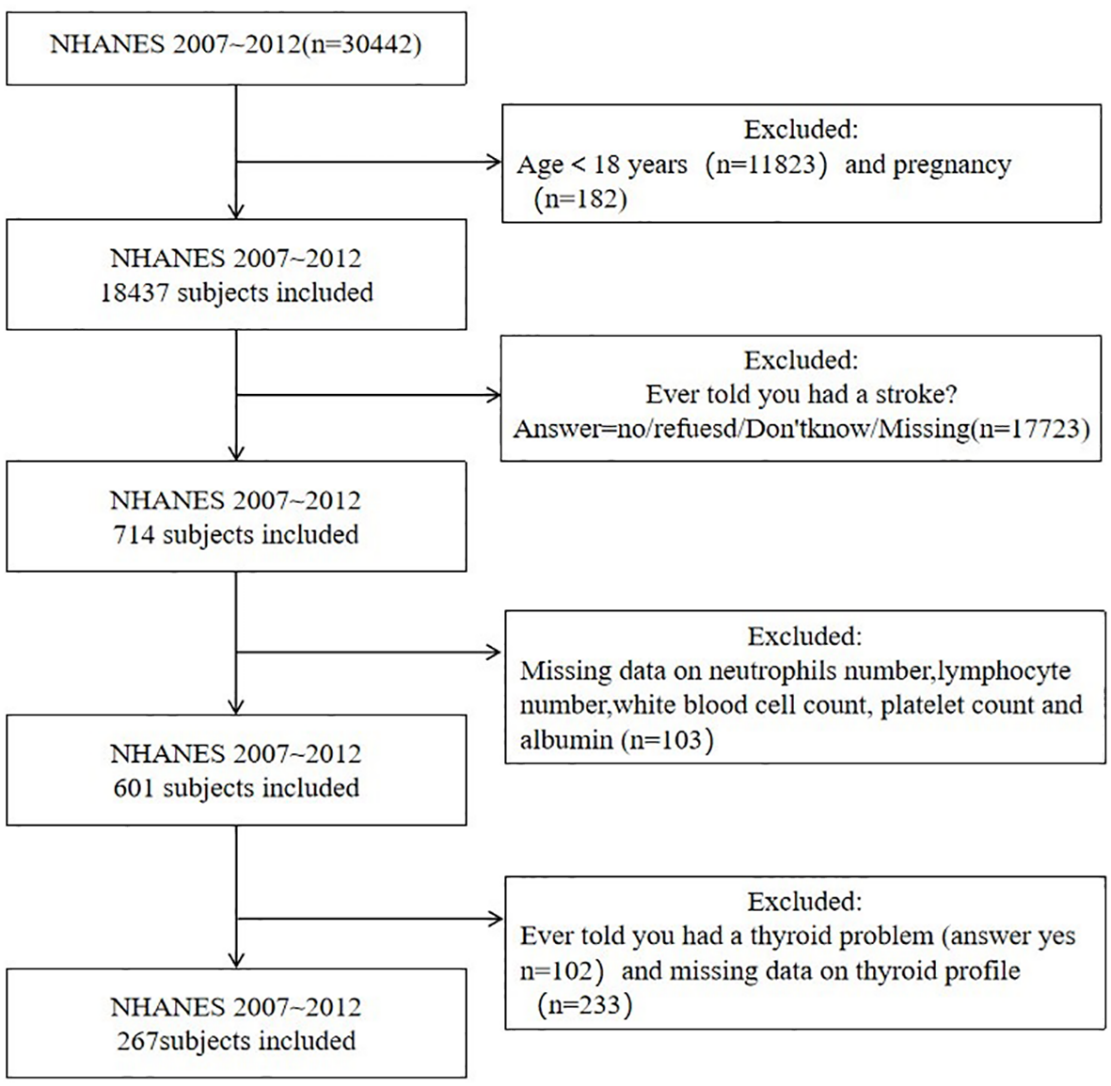
A detailed flow chart of participant recruitment.

This study identified stroke populations surveyed in the NHANES database from 2007 to 2012 as subjects. Inclusion criteria: (1) age ≥18 years old.(2) people with stroke. Exclusion criteria: (1) pregnant people.(2) people with thyroid disease.(3) people with thyroid hormone and inflammation indicators missing. The enrollment process is shown in **Figure 1**. NHANES is an open access database in the United States and has been approved by the ethics review board of the National Center for Health Statistics. All study participants voluntarily participated in the examination and questionnaire after providing written informed consent. According to the inclusion and exclusion criteria, a total of 267 subjects were included in the study.

All of the NHANES data is accessible to the public and can be downloaded freely through: https://www.cdc.gov/nchs/nhanes/index.htm.

### Information acquisition

This is a retrospective cohort study. The following data were obtained from the NHANES2007-2012 database: (1) The history of stroke, thyroid disease, hypertension, heart failure, coronary heart disease, emphysema, chronic bronchitis, cancer, and smoking were obtained through questionnaire data. (2) Gender, age, race, marital status, Ratio of family income to poverty were obtained from demographic data. (3) Body Mass Index (BMI) was obtained from body measurement data. (4) Leukocyte count (1000 cells/uL), neutrophil count (1000 cells/uL), lymphocyte count (1000 cells/uL), albumin (g/L), platelet count (1000 cells/uL), T3(ng/dL), T4(ug/dL) were obtained from laboratory data.

### Measurement of T3,T4

The same assay was used for T3,T4 in all participants. Thyroid blood specimens were processed, stored and shipped to University of Washington, Seattle, WA. The Access Total T3 assay is a competitive binding immunoenzymatic assay. Sample is added to a reaction vessel with a stripping agent to dissociate T3 from the binding proteins. T3 in the sample competes with the T3 analogue coupled to biotin for anti-T3 alkaline phosphatase conjugate. Of the resulting antigen/antibody complexes, the T3 analogue/antibody complexes are bound to the streptavidin coated solid phase. Separation in a magnetic field and washing removes the sample T3/antibody complexes and other materials not bound to the solid phase. Then, the chemiluminescent substrate Lumi-Phos™ 530 is added to the vessel and light generated by the reaction is measured with a luminometer. The light production is inversely proportional to the concentration of total T3 in the sample. The amount of analyte in the sample is determined from a stored, multi-point calibration curve. Total T4 assay is similar to total T3,a sample is added to a reaction vessel with anti-thyroxine antibody, thyroxine-alkaline phosphatase conjugate, and paramagnetic particles coated with goat anti-mouse capture antibody and a stripping agent to dissociate all T4 from binding proteins. Thyroxine in the sample competes with the thyroxine-alkaline phosphatase conjugate for binding sites on a limited amount of specific anti-thyroxine antibody. Resulting antigen: antibody complexes bind to the capture antibody on the solid phase. Subsequent steps are the same as for the determination of total T3.

### Follow-up visit

The end date of follow-up was December 31, 2019, and the defined endpoint event was death.

### Statistical analysis

SPSS and R language were used for statistical analysis. The normality of measurement data was tested by shapro-WiIk test. Measurement data conforming to normality were expressed as mean ± standard deviation, and count data were expressed as percentage. Univariate analysis was performed using the Log-rank non-parametric test and Cox regression to compare the differences in overall survival between groups, and the survival curve of T3/T4 ratio was drawn. The correlation between T3/T4 ratio and WBC, NLR, SII, NPAR was analyzed by pearson correlation analysis. Factors with *p* < 0.05 in univariate analysis were included in multivariate analysis, and Cox regression model analysis was used to construct a prognostic model to obtain the independent risk factors for death of stroke survivors. The linear relationship between T3/T4 ratio and mortality was analyzed by drawing RCS curve. Subgroup analysis was used to analyze the association between T3/T4 and mortality in each subgroup, and forest plots were drawn. Finally, the mediating effect was used to analyze whether T3/T4 ratio could have a mediating effect on survival through inflammatory indicators.

## Results

### General information and overall survival of the subjects

According to the inclusion and exclusion criteria, there were 267 people, of whom 136 survived and 131 died, with an average survival time of 111.22 ± 3.19 months and a median survival time of 130 ± 11.27 months. The general information of subjects is shown in **Table 1**.

**Table 1:** General information of test subjects.

Variables	Total (n = 267)	Death (n = 131)
Gender		
Male	147 (55.06)	68 (51.91)
Female	120 (44.94)	63 (48.09)
Age,		
<60	66 (24.72)	8 (6.11)
60~69	71 (26.59)	33 (25.19)
70~79	82 (30.71)	51 (38.93)
≥80	48 (17.98)	39 (29.77)
Race		
Mexican American	21 (7.87)	3 (2.29)
Other Hispanic	18 (6.74)	7 (5.34)
Non-Hispanic White	132 (49.44)	71 (54.20)
Non-Hispanic Black	83 (31.09)	44 (33.59)
Other Race - Including Multi-Racial	13 (4.87)	6 (4.58)
Marital Status		
Married	133 (49.81)	60 (45.80)
Never married	30 (11.24)	9 (6.87)
Widowed/Divorced	104 (38.95)	62 (47.33)
Ratio of family income to poverty		
<1.3	97 (39.75)	44 (36.36)
1.3~3.5	108 (44.26)	61 (50.41)
>3.5	39 (15.98)	16 (13.22)
Hypertension		
Yes	216 (80.90)	110 (83.97)
No	51 (19.10)	21 (16.03)
Diabetes		
Yes	87 (32.58)	45 (34.35)
No	180 (67.42)	86 (65.65)
Heart failure		
Yes	49 (18.63)	26 (20.31)
No	214 (81.37)	102 (79.69)
Coronary heart disease		
Yes	45 (17.11)	28 (21.71)
No	218 (82.89)	101 (78.29)
Emphysema		
Yes	24 (9.06)	15 (11.54)
No	241 (90.94)	115 (88.46)
Chronic bronchitis		
Yes	30 (11.28)	16 (12.31)
No	236 (88.72)	114 (87.69)
cancer or malignancy		
Yes	67 (25.09)	38 (29.01)
No	200 (74.91)	93 (70.99)
Smoking		
Yes	60 (36.36)	25 (30.86)
No	105 (63.64)	56 (69.14)
BMI		
<18.5	5 (1.96)	2 (1.65)
18.5~23.99	38 (14.90)	18 (14.88)
24~28	72 (28.24)	43 (35.54)
>28	140 (54.90)	58 (47.93)

### Survival analysis and K-M curve of T3/T4 ratio

**Figure 2:**
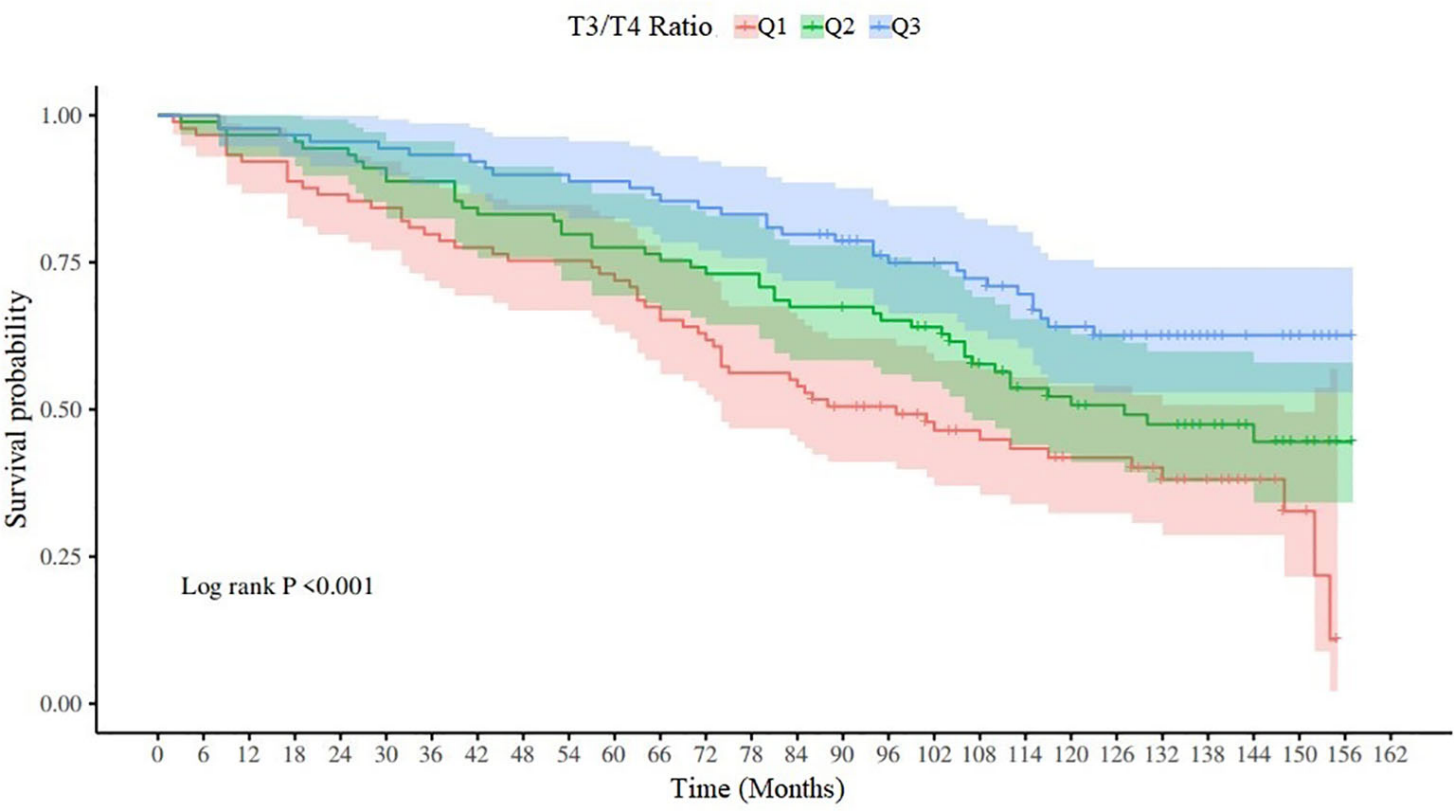
K-M survival curve of T3/T4 ratio.

According to the distribution of T3/T4 ratio, it can be divided into three quantiles, Q1 (T3/T4 ratio ≤11.69), Q2 (11.69 < T3/T4 ratio ≤14.59) and Q3 (T3/T4 ratio > 14.59). The Log-RANK test results are *x* ^2^ = 16.32, *p* < 0.001, and the K-M survival curve is shown in **Figure 2**.

### Correlation analysis of T3/T4 ratio and inflammatory indicators

The T3/T4 ratio, WBC count, NLR,SII, and NPAR were normally distributed, so pearson correlation analysis was used to analyze the correlation between T3/T4 ratio and inflammatory indicators. There was a linear relationship between T3/T4 ratio and NLR, SII, NPAR, but a weak negative correlation with NPAR (*r*=-0.31, *p* < 0.001). The specific results are shown in **Table 2**.

**Table 2:** T3/T4 ratio and correlation analysis of inflammatory indicators.

Inflammatory indicators	Value	*r*	*p*
WBC	7.38 ± 3.01	-0.06	0.316
NLR	11.33 ± 2.42	-0.22	<0.001
SII	589.63 ± 427.50	-0.16	0.01
NPAR	1.46 ± 0.80	-0.31	<0.001
T3/T4	13.32 ± 3.56	——	——

NLR, Neutrophil number/Lymphocyte number.

SII, Neutrophil number/Lymphocyte number*Platelet count.

NPAR, Neutrophil percentage (%) *100/albumin.

### Cox regression analysis and nomogram of stroke survivors

**Figure 3:**
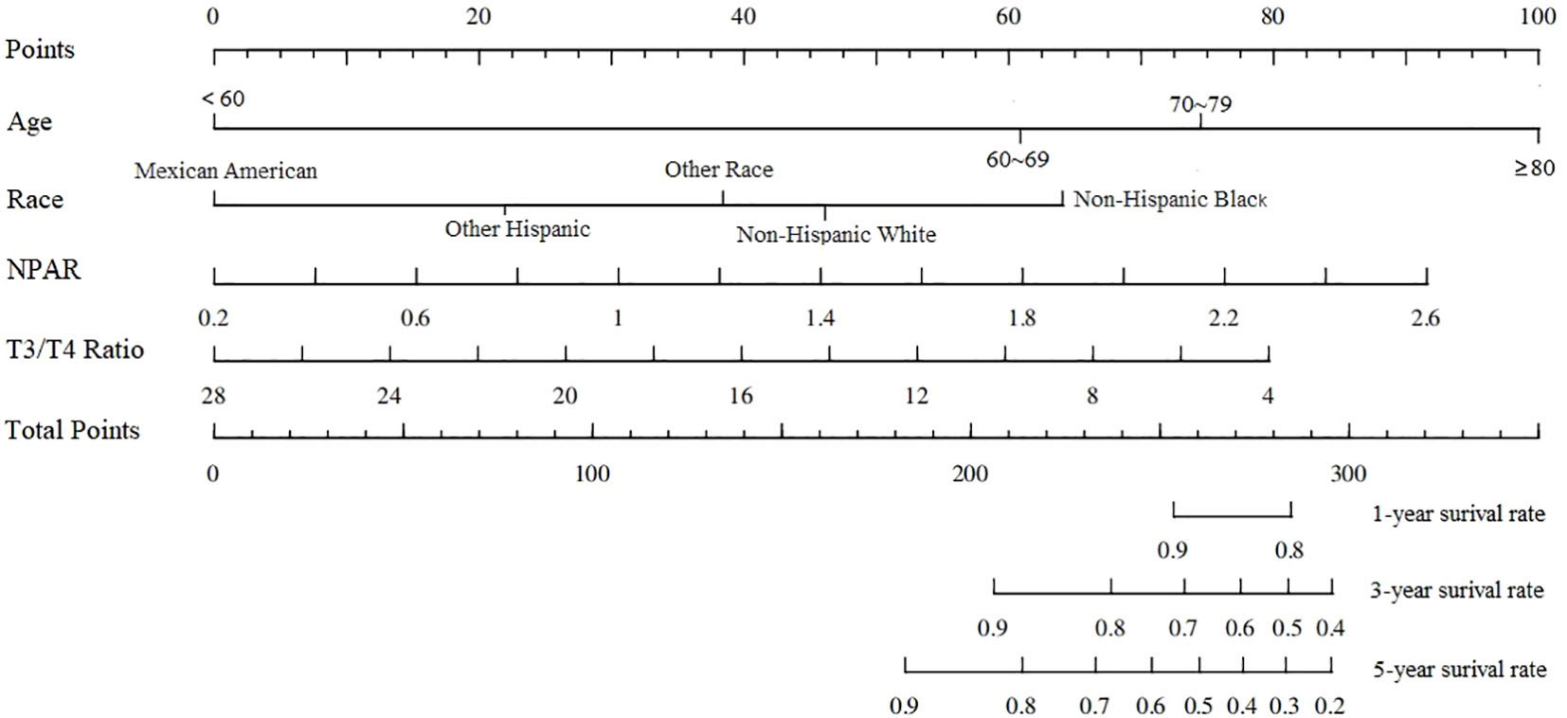
Nomogram based on multivariate Cox regression.

Univariate Cox regression analysis showed that age, marital status, race, cancer or malignancy, T3/T4 ratio and NPAR were associated with all-cause mortality in stroke survivors. The significant indicators identified in the univariate analysis were subjected to stepwise regression analysis and subsequently to multivariate COX regression analysis. Multivariate COX analysis regression analysis showed that age ≥60 years, race of non-Hispanic black, T3/T4 ratio, and NPAR were independent risk factors for all-cause mortality in stroke survivors; the specific results are shown in **Table 3**. The nomogram based on the results of multivariate Cox regression analysis is shown in **Figure 3**.

**Table 3:** Results of univariate and multivariate Cox regression for stroke survivors.

Variables	Univariate Cox regression		multivariate Cox regression	
	*p*	*HR (95%CI)*	*p*	*HR (95%CI)*
Gender				
Male		1.00 (Reference)		
Female	0.350	1.18 (0.84 ~ 1.66)		
Age,				
<60		1.00 (Reference)		1.00 (Reference)
60~69	<.001	4.69 (2.17 ~ 10.17)	<.001	4.37 (2.01 ~ 9.50)
70~79	<.001	7.54 (3.57 ~ 15.91)	<.001	6.07 (2.85 ~ 12.95)
≥80	<.001	14.16 (6.58 ~ 30.47)	<.001	11.24 (5.12 ~ 24.68)
Race				
Mexican American		1.00 (Reference)		1.00 (Reference)
Other Hispanic	0.175	2.55 (0.66 ~ 9.89)	0.442	1.71 (0.44 ~ 6.67)
Non-Hispanic White	0.012	4.43 (1.39 ~ 14.07)	0.063	3.05 (0.94 ~ 9.87)
Non-Hispanic Black	0.009	4.80 (1.49 ~ 15.45)	0.010	4.69 (1.45 ~ 15.21)
Other Race - Including Multi-Racial	0.033	4.52 (1.13 ~ 18.09)	0.199	2.52 (0.62 ~ 10.28)
Marital Status,				
Married		1.00 (Reference)		
Never married	0.125	0.58 (0.29 ~ 1.17)		
Widowed/Divorced	0.008	1.62 (1.13 ~ 2.31)		
Ratio of family income to poverty				
<1.3		1.00 (Reference)		
1.3~3.5	0.078	1.42 (0.96 ~ 2.09)		
>3.5	0.599	0.86 (0.48 ~ 1.52)		
BMI				
<18.5		1.00 (Reference)		
18.5~23.99	0.720	1.31 (0.30 ~ 5.64)		
24~28	0.464	1.70 (0.41 ~ 7.04)		
>28	0.990	1.01 (0.25 ~ 4.15)		
Hypertension				
No		1.00 (Reference)		
Yes	0.132	1.43 (0.90 ~ 2.29)		
Diabetes				
No		1.00 (Reference)		
Yes	0.581	1.11 (0.77 ~ 1.59)		
Heart failure				
No		1.00 (Reference)		
Yes	0.333	1.24 (0.80 ~ 1.90)		
Coronary heart disease				
No		1.00 (Reference)		
Yes	0.120	1.39 (0.92 ~ 2.12)		
Emphysema				
No		1.00 (Reference)		
Yes	0.211	1.41 (0.82 ~ 2.41)		
Chronic bronchitis				
No		1.00 (Reference)		
Yes	0.813	1.07 (0.63 ~ 1.80)		
cancer or malignancy				
No		1.00 (Reference)		
Yes	0.042	1.48 (1.01 ~ 2.17)		
Smoking				
No		1.00 (Reference)		
Yes	0.117	0.69 (0.43 ~ 1.10)		
T3/T4 ratio	<.001	0.87 (0.82 ~ 0.92)	0.014	0.92 (0.87 ~ 0.98)
FT3/FT4 ratio	<.001	0.56 (0.45 ~ 0.71)		
NPAR	<.001	4.48 (2.35 ~ 8.52)	0.009	2.50 (1.26 ~ 4.99)

### RCS curve and the best cut-off value

**Figure 4:**
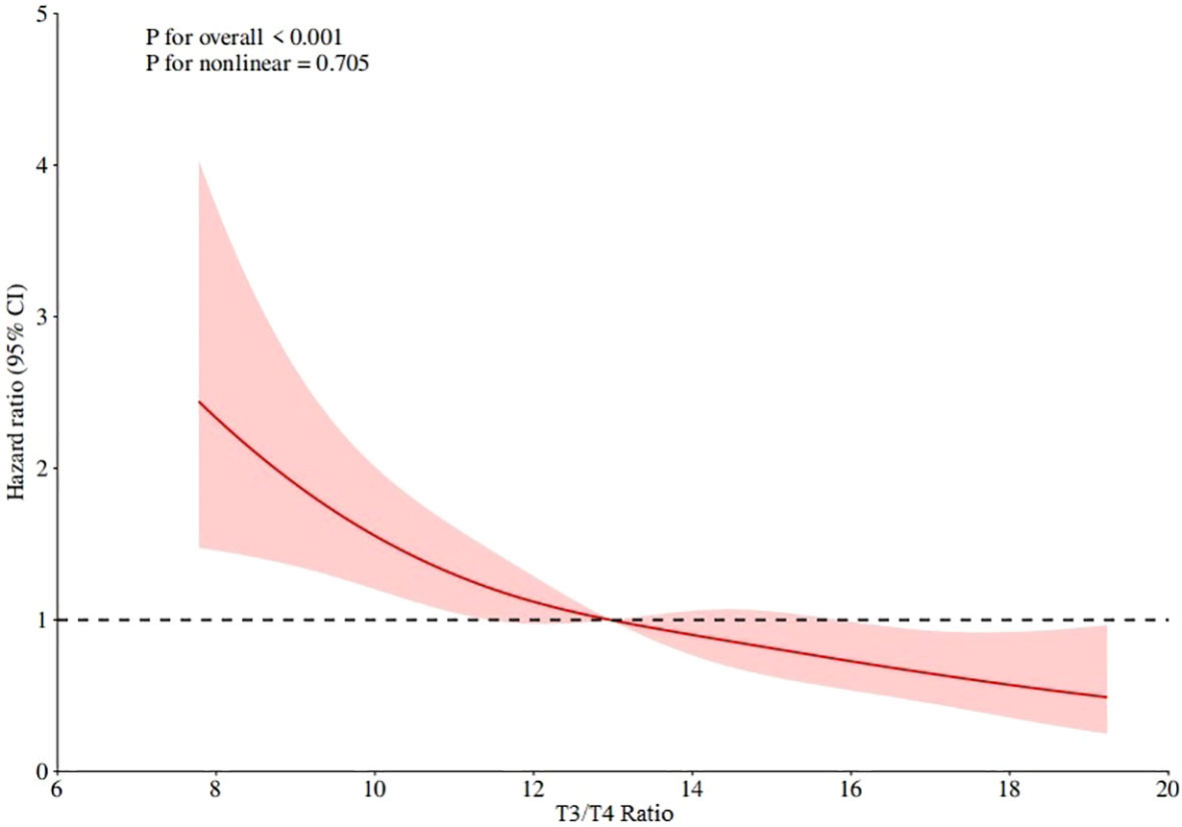
RCS curve of T3/T4 ratio and all-cause mortality in all subjects.

The RCS curve of T3/T4 ratio and mortality was drawn by Cox regression model to determine the linear relationship and the best order cut-off value. The RCS curve results are shown in **Figure 4**, which shows that *p* for overall < 0.001, *p* for nonlinear=0.705, indicating that the T3/T4 ratio has a linear relationship with mortality, the lower the T3/T4 ratio, the higher the mortality, and the best cut-off value is 12.97.

### Subgroup analysis and forest plots

**Figure 5:**
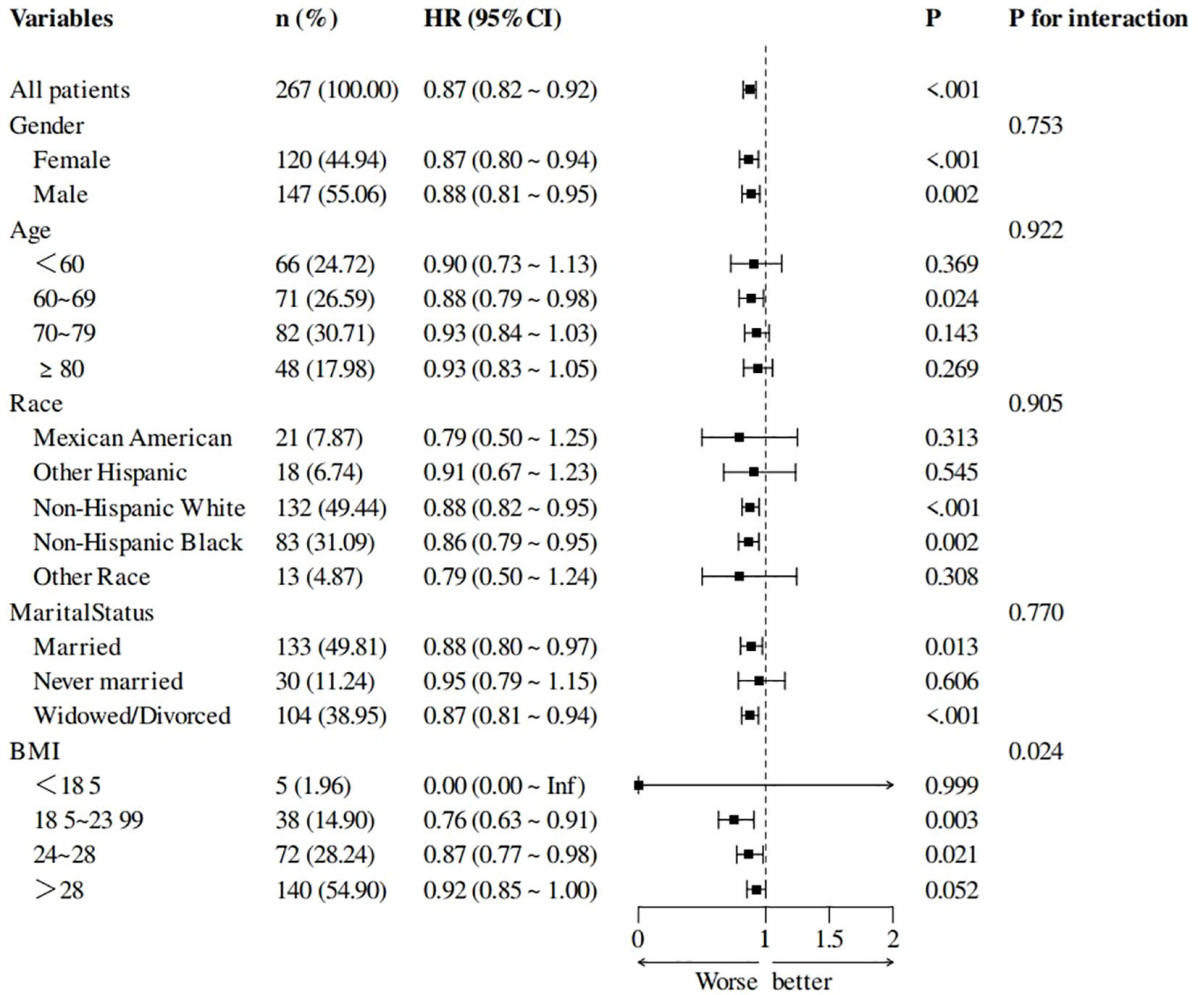
Subgroup analysis of T3/T4 ratio and all-cause mortality forest plots, adjusted for age, race, and NPAR.

Subgroup analyses showed the interaction effect only in the BMI subgroup. T3/T4 ratio is more likely to affect the survival of stroke survivors with BMI 18.5~28. The results of subgroup analyses and forest plots are shown in **Figure 5**.

### Analysis of the mediating effect of T3/T4 ratio, NPAR and survival time

**Figure 6:**
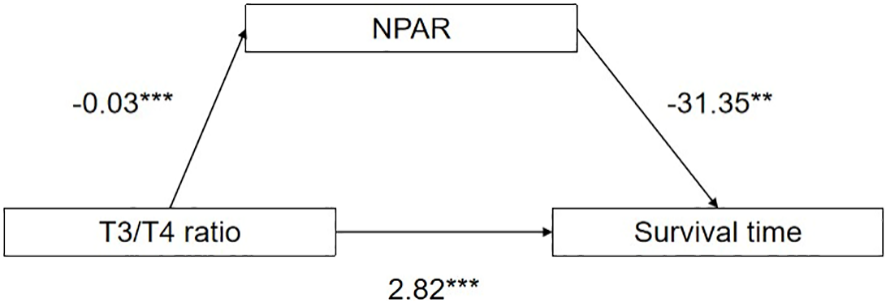
Path coefficient plots for T3/T4 ratio, NPAR, and survival time.

In order to explore the underlying mechanism of the effect of T3/T4 ratio on survival time, NPAR was further introduced into the structural equation model as a mediating variable. The SPSS macro program Process Model 4 was used to test the mediating effect, and the bootstrap method was used to verify and analyze the mediating effect of NPAR on T3/T4 ratio and survival time. The path coefficients of the three variables of NPAR in T3/T4 ratio and survival time are shown in **Figure 6**. The breakdown table of total effect, direct effect and mediating effect is shown in **Table 4**. Both the upper and lower limits of the bootstrap 95% CI for the mediation effect of T3/T4 ratio on survival time and NPAR did not include 0, indicating that T3/T4 ratio not only directly affected survival time, but also mediated the effect of NPAR on survival time. The direct effect (2.82) and mediating effect (0.80) accounted for 78.45% and 21.55% of the total effect, respectively.

**Table 4:** Breakdown table of total effect, direct effect and mediation effect.

	Size of effect	se	LLCI	ULCL	Percentage of effect
Total effect	3.62	0.77	2.11	5.13	
Direct effect	2.82	0.78	1.7	4.77	78.45%
Mediation effect	0.80	0.19	0.03	0.77	21.55%

## Discussion

Our study highlights the need to monitor thyroid function in stroke survivors by analyzing the association between T3/T4 ratio and all-cause mortality among participants in the NHANES database from 2007 to 2012. Q1,Q2 and Q3 of the ratio of T3/T4 showed significant differences on the K-M curve, and the survival time decreased with the decrease of the ratio of T3/T4. Multivariate Cox regression analysis showed that after adjusting for age, race and other factors, low T3/T4 ratio was an independent risk factor for all-cause mortality, and the risk of death increased by 8% with a decrease of one unit. In the correlation analysis with WBC, NLR, SII, and NPAR, T3/T4 ratio was only weakly negatively correlated with NPAR.

Based on statistical results and previous studies of stroke and thyroid hormones we chose the T3/T4 ratio instead of FT3/FT4. (1) FT3/TF4 showed between-group differences in one-way COX regression analyses, but T3/T4 was chosen over FT3/FT4 after stepwise regression was performed, which represents the results of this study only, and sample differences may exist. (2) Two large sample size studies on stroke and thyroid hormone (including T3,T4,FT3,FT4,TSH) levels showed that low T3 levels predicted a poor functional prognosis after ischemic stroke in adults, and that higher T4 levels and lower T3 levels were also associated with higher clinical severity of stroke on admission (9, 10).

Many factors affect the conversion of T4 to T3, but the most important is the action of deiodinase (11). Different deiodinases have different tissue distribution, substrate affinity and physiological effects. Based on their deiodination characteristics and distribution sites, they are classified as type I (DIO1), type II (DIO2), and type III (DIO3) deiodinases (12). DIO1 and DIO2 activate T4 by removing an iodine atom from the outer loop of T4 to form T3. On the other hand, DIO3 inactivates T3 and T4 by removing iodine atoms from the inner ring to generate diiodothyronine (T2) and reverse T3, respectively (13).

There is an interaction relationship between thyroid hormone level and inflammatory immune response. Thyroid hormone as a source of iodine plays an important role in the myeloperoxidase-hydroperoxide-halide antibacterial system of neutrophils (14). As thyroid hormone is degraded, the concentration of iodine in neutrophils increases, thereby enhancing the bactericidal activity of neutrophils (15). In addition, bacterial killing by neutrophils can be mediated by the production of reactive oxygen species through the NADPH oxidase system (16). Circulating thyroid hormone levels affect reactive oxygen species (ROS) production by stimulated neutrophils: hyperthyroidism leads to increased ROS production by ex vivo stimulated neutrophils compared to euthyroid controls, whereas hypothyroidism has the opposite effect, limiting ROS production by neutrophils (17). Cytokines are autocrine and paracrine signaling peptide messengers that function in complex networks that function in the immune system and coordinate inflammatory responses. At present, the mainstream view is that interleukin (IL) β, tumor necrosis factor α (TNF-α), especially IL-6 play a role in the pathogenesis of ESS (18). An inverse correlation between IL-1, IL-6, IL-10, and C-reactive protein and T3 and T4 concentrations has been reported in different patient populations (19).

Abnormal thyroid hormones may be associated with poor outcomes in patients with acute stroke. Zhang et al. (20) found that the percentage of patients with low T3 showing good neurological improvement as measured by National Institute of Health stroke scale (NIHSS) and Modified Rankin Scale (mRS) was significantly lower compared with patients with normal T3, suggesting that low T3 is associated with worse neurological outcome and that the severity of low T3 may be a predictor of functional improvement in acute ischemic stroke. Li et al. (9) found that the association between low T3 levels and poor prognosis after acute stroke did not apply to all age groups, and that lower total T3 concentrations were independently associated with poor functional prognosis in the older age group (≥65 years), so that the assessment of T3 levels is more relevant in the elderly population. Alevizaki et al. (21) evaluated 737 patients with first acute stroke, the 1-year mortality of low T3 cases on the second day of admission was 27.34%, and the 1-year mortality of normal T3 cases was 19.37%, and Cox regression analysis showed that T3 level was a significant predictor of 1-year mortality. From a clinical perspective, it is noteworthy that most studies used normal T3 concentration as the reference standard. Wang et al. (22) found that T3 <1.03 nmol/L was the cutoff value for 90-day prognosis in stroke patients, with an AUC of 0.70, sensitivity of 83%, and specificity of 60%, and that the combination of the NHISS score and age could contribute to a better prognostic assessment. Based on the results of their study, the cut-off value of T3 was 66.88 ng/dL, the range of T4 was 5.40~8.31 ug/dL, and the cut-off value of the estimated T3/T4 ratio was 8.04~12.39, which was close to the cut-off value of Q1 group. However, the T3/T4 grouping method and the cut-off value obtained according to RCS curve in our study need more research to verify the clinical applicability.

In addition to the effects of disease, thyroid hormone conversion may be associated with aging. As TSH levels increased, the FT3/FT4 ratio increased until age 40, but this increase in difference did not occur in the older population, which may reflect that the conversion of T4 to T3 decreases with age, possibly as part of the aging process (23). Ostan et al. (24) analyzed 672 cases of elderly Italian (age range: 52~113 years), the results show that FT3 level and FT3/FT4 ratio decrease while FT4 and TSH increase in an age-dependent manner. Moreover, they found that higher FT4 levels and lower FT3/FT4 ratios were associated with impaired functional status and increased mortality in semi-supercentenarians. Arosio et al. (25) first found an association between thyroid hormones and the Frailty Index (FI) in long-lived individuals, underpinning the important role of the thyroid gland in the aging process and longevity. Their study found that in all centenarians, FI was negatively correlated with FT3/FT4 (r = -0.344, *p* < 0.001), and in centenarians with hormone levels in the normal range, FI was similarly negatively correlated with FT3/FT4 (r = -0.264, *p* = 0.002). Furthermore, in the elderly population with normal thyroid function, low FT3/FT4 was a better indicator of low muscle mass and impaired physical function than FT3 or FT4 alone (26). The age-related decrease in the FT3/FT4 ratio could be due to a decline in 5,-deiodinase activity (27). Centenarians and semisupercentenarians with a relatively high FT3/FT4 ratio are probably able to preserve DIO1 activity, likely maintaining a good hormonal negative feedback (24). In particular, preserving thyroid hormone signaling by better maintaining local T3 concentrations through conversion of appropriate peripheral T4 to T3 may play an important role in ensuring longevity and healthy aging (27).

NPAR also has a predictive role in stroke patients. A retrospective study involving 364 subjects found that higher NPAR independently predicted ICU admission for patients with ischemic stroke and became a robust independent predictor of ICU admission for patients with ischemic stroke, exceeding the predictive performance of NLR (28). In addition, NPAR may be a marker for predicting stroke recurrence. Yang et al. (29) found that NPAR was independently associated with recurrence within 3 months in patients with first-ever acute ischemic stroke, and showed the largest area under the ROC curve when compared with other markers, indicating that NPAR may be a more effective biomarker for predicting recurrence in patients with acute ischemic stroke than albumin level, neutrophil percentage and NLR. However, no studies have found the correlation between thyroid hormone levels and NPAR, or the combination of the two to predict the prognosis of the disease.

## Limitations

Our study has certain limitations. First, the NHANES database had thyroid-hormone levels only for the period from 2007 through 2012, which prevented the study from having a very large sample size. Second, much of the data comes from questionnaires, which may have subjective bias. Then, some medications (such as thyroid hormone preparations, methimazole, propylthiouracil, etc) can affect thyroid hormone levels, and due to database limitations, it was not possible to exclude the effects of these medications on thyroid hormones. Finally, factors associated with mortality, such as stroke severity at the time of stroke, lesion location, and rehabilitation, were not available from the database.

## Conclusions

Monitoring thyroid function in stroke survivors has an important role in all-cause mortality. After adjusting for age, race and other factors, T3/T4 ratio was an independent risk factor for all-cause mortality in stroke survivors, especially in those BMI of 18.5~28. T3/T4 ratio may play a mediating effect on survival time through NPAR level. The optimal frequency of thyroid function monitoring and the effect of thyroid hormone replacement therapy still need to be clarified by high-quality studies.

## Data Availability

All of the NHANES data is accessible to the public and can be downloaded freely through: https://www.cdc.gov/nchs/nhanes/index.htm.
